# A Case of Larval Oriental Eye Worm, Thelazia callipaeda, Infection Overlooked in Routine Ophthalmic Practice

**DOI:** 10.7759/cureus.70717

**Published:** 2024-10-02

**Authors:** Naoki Uchida, Yumare Wada, Ayaka Shimada, Akiko Harano, Sho Ichioka, Hitoshi Otsuki, Masaki Tanito

**Affiliations:** 1 Department of Ophthalmology, Shimane University Faculty of Medicine, Izumo, JPN; 2 Division of Medical Zoology, Department of Microbiology and Immunology, Faculty of Medicine, Tottori University, Yonago, JPN

**Keywords:** conjunctivitis, ophthalmic diagnosis, oriental eyeworm, thelazia callipaeda, zoonotic infection

## Abstract

*Thelazia callipaeda*, the oriental eyeworm, is a zoonotic parasite that primarily infects the eyes, causing symptoms such as foreign body sensation, tearing, and itching. It is transmitted by fruit flies like *Phortica okadai *and commonly affects dogs and cats. This case report describes an 18-year-old woman who presented with complaints of “a worm in her left eye.” Despite two visits to an ophthalmology clinic, she was initially diagnosed with conjunctivitis. The patient’s history of dog ownership raised the possibility of zoonotic transmission; however, the worm was not detected during clinical examinations. Eventually, the patient removed the worm herself, and it was identified as the larval stage of *T. callipaeda*. This case emphasizes the importance of considering *T. callipaeda *in patients with nonspecific conjunctivitis symptoms, especially those with a history of contact with animals. It highlights the challenges in diagnosing this rare infection due to its nonspecific presentation and the small size of the larvae. Clinicians should be aware of this possibility and conduct thorough examinations, including double eversion of the eyelids, to avoid misdiagnosis and ensure appropriate treatment.

## Introduction

*Thelazia callipaeda*, also known as the oriental eyeworm, is a zoonotic parasite primarily found in South Asia, Russia, and China [1]. It is transmitted by fruit flies, particularly *Phortica okadai*. The prevalence of thelaziasis is associated with the breeding season of these flies, typically from early summer to early autumn (June to September in Japan) [1]. Dogs and cats are the most common intermediate hosts, serving as sources of infection for humans [2]. In Japan, several cases of *T. callipaeda* infection have been reported [3-5]. This parasite causes ocular symptoms such as tearing, foreign body sensation, itching, conjunctivitis, and conjunctival follicular hypertrophy. In more severe cases, keratitis and corneal ulcers may develop [6]. Treatment in humans primarily involves the mechanical removal of the parasites. However, detecting *T. callipaeda* can be challenging, particularly in larval infections [6]. This paper presents the case of an 18-year-old woman who visited a clinic twice and complained of “a worm in her left eye” but was diagnosed with conjunctivitis on both occasions without the worm being discovered. This case highlights the importance of considering *T. callipaeda *as a differential diagnosis in cases of non-specific conjunctivitis.

## Case presentation

On September 6, an 18-year-old woman noticed “a worm in her left eye” while looking in the mirror. She had no remarkable past medical history and had been living with a dog at her parents' house for the past year. On September 20, she visited an ophthalmology clinic for the first time with the chief complaint of “a worm in her left eye.” A slit-lamp examination by an ophthalmologist revealed no worm, and she was diagnosed with conjunctivitis and prescribed 0.1% fluorometholone eye drops. On October 1, she again noticed the worm in her left eye and returned to the clinic on October 4. Once more, the ophthalmologist did not find the worm and suspected bacterial conjunctivitis, prescribing 1.5% levofloxacin eye drops.

**Figure 1:**
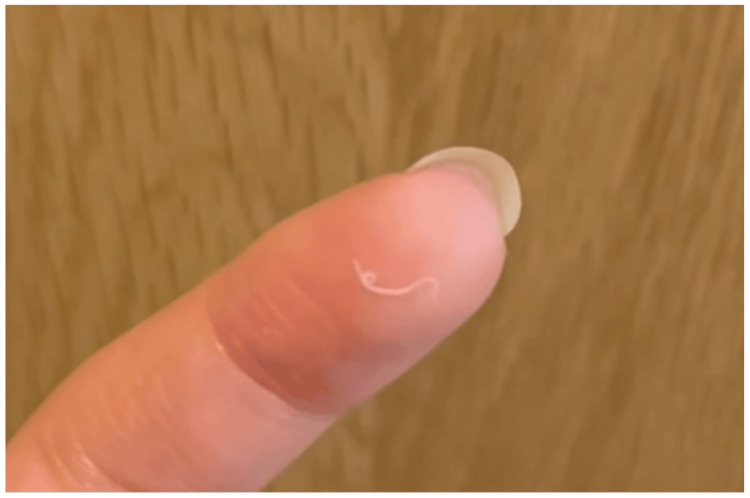
A snapshot of the “worm” captured from the video provided by the patient.

**Figure 2:**
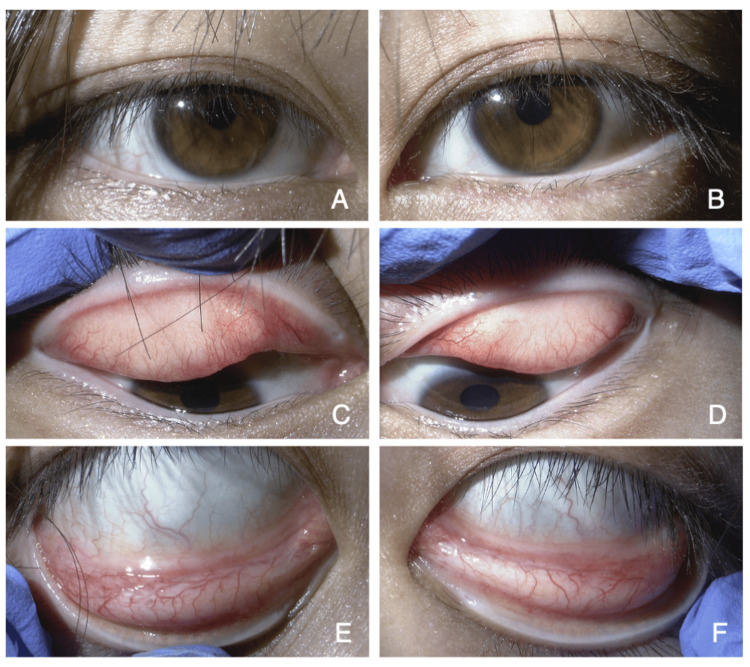
Slit-lamp examination findings of the right (A, C, E) and left (B, D, F) eyes. At the initial visit to our hospital, no signs of inflammation or remaining “worm” are observed in the eyelids (A, B), superior (C, D), and inferior (E, F) conjunctiva of both eyes.

On October 6, the patient found the worm while looking in the mirror at home and removed it with her fingers. She recorded a video of the worm using her smartphone (Figure 1, Video 1) and stored the worm in physiological saline. On October 8, she revisited the clinic, presenting the removed worm and the video to the ophthalmologist. Suspecting an eyeworm infection, the ophthalmologist referred her to the Department of Ophthalmology at Shimane University Hospital for further evaluation. At the initial visit to Shimane University Hospital, her uncorrected visual acuity was 1.2 on a decimal visual acuity chart in both eyes. A slit-lamp examination showed a clear cornea, a deep anterior chamber without abnormalities, and no evidence of cataracts in either eye (Figures 2A and 2B). The fundus was also normal. Despite a thorough examination of the conjunctival fornix by everting both eyelids, no foreign material or remaining worm was detected (Figure 2C-F).

**Video 1:** Video of the “worm” recorded by the patient.

**Figure 3:**
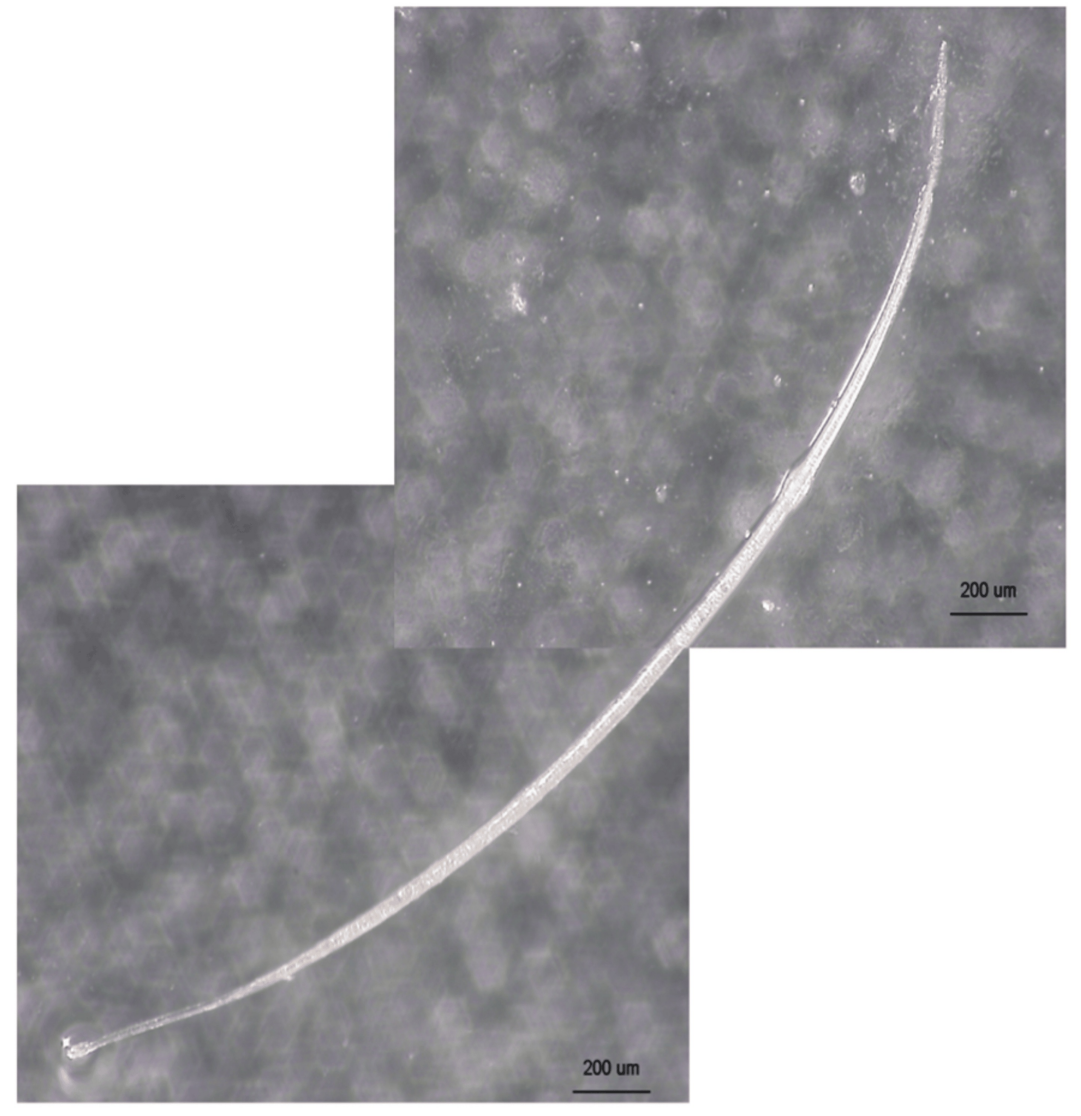
Stereomicroscopic view of the “worm.”

The worm brought by the patient was preserved in formalin and examined under a stereomicroscope by a parasitologist (Figure 3). Considering features such as size (approximately 3.2 mm), absence of internal reproductive structures, and the location of infection (the conjunctival sac), the nematode was identified as a larval stage of *T. callipaeda*.

On October 23, the patient was informed that the parasite was most likely *T. callipaeda* and that no further treatment was necessary following its removal. She was also advised to have her dog examined by a veterinarian, as dogs are common hosts of this parasite.

## Discussion

Previous cases have reported situations where *T. callipaeda* was not detected during preoperative examinations and was discovered only during cataract surgery [7]. In those instances, the failure to detect the parasite was attributed to inadequate examination of the conjunctival sac and communication difficulties due to language barriers [7]. In the present case, the patient repeatedly reported the “presence of a worm in her left eye,” but it was not detected during two separate visits. These cases highlight the difficulty in accurately diagnosing *T. callipaeda* infection in routine ophthalmic care.

The adult *T. callipaeda *typically measures 10-15 mm, while the parasite in this case was only about 3.2 mm. *T. callipaeda* is known to be transmitted by flies of the Phortica variegata species and undergoes two molts to become an adult worm [8,9]. However, little literature details the morphology of the larval stages, making it challenging to identify the worm based on appearance alone. In this case, the parasitologist identified the larval stage of *T. callipaeda *through a comprehensive assessment of characteristics, including shape, size, and the removal site of the parasite. This patient lived in western Japan, where *T. callipaeda *infections are most commonly reported.

Several factors contributed to the diagnostic difficulties in this case. The rarity of *T. callipaeda* infections could lead to misdiagnosis, even with a specific complaint. Additionally, the parasite’s transparency and small size in the larval stage might have hindered detection. Moreover, the patient did not experience vision loss, redness, or significant abnormalities in the conjunctiva or cornea, leading to nonspecific clinical findings. This could result in a default diagnosis of conjunctivitis, with common treatments like steroids and antibiotic eye drops.

Given that *T. callipaeda* is a rare parasite with a nonspecific clinical presentation, it can often be overlooked. However, considering the patient’s history of dog ownership and her clear statement about the “presence of a worm in her eye,” more thorough examination techniques, such as double eversion of the eyelids and fluorescein staining, might have led to an earlier discovery. Therefore, when making a differential diagnosis, it is essential to consider the possibility of *T. callipaeda* and not dismiss the patient’s claims.

## Conclusions

This case highlights the importance of considering *T. callipaeda* infection in patients presenting with conjunctivitis symptoms, especially when there is a history of dog ownership and specific complaints, and emphasizes the need for conducting appropriate examinations.
